# Glial Growth Factor 2 Regulates Glucose Transport in Healthy Cardiac Myocytes and During Myocardial Infarction via an Akt-Dependent Pathway

**DOI:** 10.3389/fphys.2019.00189

**Published:** 2019-03-27

**Authors:** Shanell Shoop, Zahra Maria, Allison Campolo, Nabil Rashdan, Dominic Martin, Pamela Lovern, Véronique A. Lacombe

**Affiliations:** ^1^Department of Physiological Sciences, Oklahoma State University, Stillwater, OK, United States; ^2^Department of Biochemistry and Molecular Biology, Oklahoma State University, Stillwater, OK, United States; ^3^Harold Hamm Diabetes Center, University of Oklahoma, Oklahoma City, OK, United States

**Keywords:** neuregulin, myocardial infarction, glucose transporters, AS160, heart

## Abstract

Neuregulin (NRG), a paracrine factor in myocytes, promotes cardiac development via the ErbB receptors. NRG-1β also improves cardiac function and cell survival after myocardial infarction (MI), although the mechanisms underlying these cardioprotective effects are not well elucidated. Increased glucose uptake has been shown to be cardio-protective during MI. We hypothesized that treatment with a recombinant version of NRG-1β, glial growth factor 2 (GGF2), will enhance glucose transport in the healthy myocardium and during MI. Cardiac myocytes were isolated from MI and healthy adult rats, and subsequently incubated with or without insulin or GGF2. Glucose uptake was measured using a fluorescent D-glucose analog. The translocation of glucose transporter (GLUT) 4 to the cell surface, the rate-limiting step in glucose uptake, was measured using a photolabeled biotinylation assay in isolated myocytes. Similar to insulin, acute *in vitro* GGF2 treatment increased glucose uptake in healthy cardiac myocytes (by 40 and 49%, respectively, *P* = 0.002). GGF2 treatment also increased GLUT4 translocation in healthy myocytes by 184% (*P* < 0.01), while ErbB 2/4 receptor blockade (by afatinib) abolished these effects. In addition, GGF2 treatment enhanced Akt phosphorylation (at both threonine and serine sites, by 75 and 139%, respectively, *P* = 0.029 and *P* = 0.01), which was blunted by ErbB 2/4 receptor blockade. GGF2 treatment increased the phosphorylation of AS160 (an Akt effector) by 72% (*P* < 0.05), as well as the phosphorylation of PDK-1 and PKC (by 118 and 92%, respectively, *P* < 0.05). During MI, cardiac GLUT4 translocation was downregulated by 44% (*P* = 0.004) and was partially rescued by both *in vitro* insulin and GGF2 treatment. Our data demonstrate that acute GGF2 treatment increased glucose transport in cardiac myocytes by activating the ErbB 2/4 receptors and subsequent key downstream effectors (i.e., PDK-1, Akt, AS160, and PKC). These findings highlight novel mechanisms of action of GGF2, which warrant further investigation in patients with heart failure.

## Introduction

Heart failure is a complex syndrome that develops over months to years and affects approximately 5 million people in the United States (Writing Group et al., 2016). Recent investigations have implicated chronic HF and MI in the development of adverse metabolic alterations, including impaired glucose uptake and utilization (Murray et al., 2006; Penuelas et al., 2007). As the heart has a greater rate of glucose utilization than other tissues, impaired glucose utilization as a result of decreased glucose uptake could be a major pathophysiologic factor of contractile dysfunction during HF and MI (Riehle and Abel, 2016). The translocation of GLUT4, the major insulin-sensitive GLUT isoform, from intracellular vesicles to the cell surface (active site) is the rate-limiting step in glucose uptake, and is regulated by insulin-dependent and –independent processes (Lacombe et al., 2003; Waller et al., 2015). In light of the fact that glucose uptake is crucial to proper cardiac function, metabolic therapy has emerged as a promising new therapeutic avenue for patients affected by HF.

Neuregulins are signaling proteins that facilitate cell–cell interactions in many tissues, including the nervous system and the heart (Falls, 2003; Wadugu and Kühn, 2012). As a member of the EGF family, NRG-1 initiates proliferation, differentiation and survival in myocytes (Holmes et al., 1992; Marchionni et al., 1993). All NRG isoforms that contain the EGF-like domain undergo alternative splicing, which yields α or β variants. Of the two variants, the β variant is considered to be the most active. In addition, only the β isoform is biologically active on cardiac myocytes (Marchionni et al., 1993). Importantly, NRG-1β signaling, via its ErbB2 and ErbB4 receptors, is crucial for proper function of the adult heart (Marchionni et al., 1993; Tania et al., 2014). In addition, blunted expression of ErbB2 or ErbB4 receptors *in vivo* led to mortality *in utero* in knockout models, due to the failure of cardiac development of the endocardial cushions and trabeculae (Gassmann et al., 1995; Lee et al., 1995; Meyer and Birchmeier, 1995).

Pre-clinical and clinical studies have demonstrated the beneficial therapeutic effects of two forms of recombinant NRG-1β on cardiac function (Gao et al., 2010; Xu et al., 2010; Brittain et al., 2013; Lenihan et al., 2013). Recombinant NRG-1β (rhNRG-1), comprised of solely the EGF-like domain, has been evaluated as a potential therapeutic agent for MI, ischemia/reperfusion injury and diabetic cardiomyopathy (Iaci et al., 2010; Galindo et al., 2014). GGF2 is a full-length splice variant of the NRG-1 gene (also known as cimaglermin alfa or NRG-1β3), which has been investigated as a novel therapeutic strategy for cardiovascular diseases (Buonanno and Fischbach, 2001). For instance, GGF2 administered to patients with symptomatic HF improved ejection fraction at days 28 and 90 (Brittain et al., 2013; Lenihan et al., 2013). Importantly, a single intravenous dose of GGF2 reached similar efficacy as a 10 day intravenous infusion of rhNRG-1 (Brittain et al., 2013; Lenihan et al., 2013). Yet, the underlying mechanisms by which GGF2 improves cardiac function in these studies remain incompletely understood. Interestingly, it has been reported that NRG-1β stimulates glucose transport in skeletal muscle cells via a PI3K-dependent pathway, activating known downstream effectors such as PDK-1, Akt, and protein kinase C (PKC-ζ) (Cantó et al., 2006). Whether GGF2 has similar metabolic effects in the heart is unknown. Therefore, we hypothesized that treatment with the full-length recombinant NRG-1β3 GGF2 will enhance glucose transport in the healthy myocardium and during MI.

## Materials and Methods

### Animals

All the procedures of this study were approved by the Oklahoma State University Institutional Animal Care and Use Committee (ACUP# VM 12-3). MI was induced by ligating the left anterior descending coronary artery in adult male Wistar anesthetized rats (Charles River). Anesthesia was performed using a cocktail of ketamine (70–100 mg/kg, IP) and xylazine (6–10 mg/kg, IP). Animals were intubated and positive pressure ventilation was provided by a ventilator. The left anterior descending coronary artery was permanently ligated using 7-0 silk suture. After completion of the procedure (in approximately 60 min), the animals were placed in a heated oxygen chamber, and subsequently transferred to their holding cages for recovery. Analgesics (e.g., buprenorphine and carprofen) were administered subcutaneously before and after surgery, as required.

Transthoracic echocardiographic examination was performed in rats lightly anesthetized with isoflurane (1–1.5% isoflurane in O_2_) at 9 and 14 days after the induction of MI, as previously described by our group (Dirksen et al., 2007; Lacombe et al., 2007; Ware et al., 2011). Ventricular structure and function were assessed by two-dimensional cine loops of a long-axis view and of a short-axis view at mid-level of the papillary muscles, as well as M-mode loops of the short-axis view. Left ventricular (LV) ejection fraction (EF), a surrogate of systolic function, was calculated offline, as follows: EF = (LVID end-diastolic - LVID end-systolic/LVID end-diastolic) × 100%.

### Ventricular Myocyte Isolation

Fourteen days following surgery, animals were deeply anesthetized (by 5% isoflurane) and hearts were extracted. Ventricular myocytes were isolated from MI and age-matched healthy rats using a Langendorff apparatus, as previously described by our group (Waller et al., 2013, 2015; Maria et al., 2015). Ventricular myocytes were obtained by enzymatic perfusion using collagenase. The ischemic patch of the myocardium of the MI animals was discarded following digestion. Ventricular myocytes (within 2 h of isolation) were then incubated for 1 h without (basal) or with insulin (0.01 μM) or incremental concentrations of GGF2 (from 1 to 100 ng/ml, Acorda Therapeutics, USAN – cimaglermin alfa). To further investigate if GGF2 stimulates GLUT translocation via the ErbB receptors, ventricular myocytes were incubated with/without ErbB (2 and 4) blocking antibodies (Afatinib, 300 nM, Selleck Chem BIBW2992) for 1 h before treatment without (basal) or with: (1) insulin (0.01 μM); (2) GGF2 (100 ng/ml) for 1 additional hour.

### Glucose Uptake Assay

Glucose uptake was measured in isolated fresh cardiac myocytes using a non-radioactive glucose uptake cell-based assay and a fluorescent D-analog, 1-N-7-(nitrobenz-2-oxa-1, 3-diazol-4-yl) amino-2-deoxy-D-glucose (2-NBDG), following manufacturer’s recommendations (Cayman Chemical, Ann Arbor, MI, United States). The cells were plated on a 96-well plate at a density of 5,000 cells/well in glucose-free Tyrode buffer and incubated with/without GGF2 or insulin (0.01 μM) for 30 min. Subsequently, 2-NBDG (100 μM) was added to each well. After a 10-min incubation with 2-NBDG, cells were washed with PBS (200 μL/well) twice, and fluorescence was measured using a microplate reader at excitation/emission wavelengths of 535/758 nM.

### Western Immunoblotting

Ventricular myocytes were lysed with RIPA (Thermo Fisher) and Protease Inhibitor Cocktail (Sigma-Aldrich, P8340). Equal amounts of protein (5–20 μg) were resolved in an 8–12% SDS-polyacrylamide gel and electrophoretically transferred to a polyvinylidene fluoride membrane (PVDF, Bio-Rad), with subsequent immunoblotting, as previously described (Lacombe et al., 2007; Waller et al., 2011b, 2013, 2015; Ware et al., 2011; Lacombe, 2014; Maria et al., 2015). After blocking (1–5% non-fat dry milk or 2% goat serum), membranes were incubated with optimally diluted primary antibodies overnight (polyclonal rabbit anti-human GLUT4, 1:750, AbD Serotec 4670–1704; monoclonal rabbit anti-mouse total Akt, 1:1000, Cell Signaling 4061; monoclonal rabbit anti-human phosphorylated Akt s473, 1:1000, Cell Signaling 4060; monoclonal rabbit anti-mouse phosphorylated Akt Th308, 1:1000, Cell Signaling 2965; monoclonal rabbit anti-human total AS160, 1:1000, Cell Signaling 2670 and polyclonal rabbit anti-human phosphorylated AS160, 1:1000, Cell Signaling 9611; polyclonal rabbit anti-human PDK-1, 1:1000, Cell Signaling 3062; Monoclonal rabbit anti-human phosphorylated PDK-1 S241, 1:1000, Cell Signaling 3438; Monoclonal Rabbit IgG anti-human PKCζ Th410, 1:500, Cell Signaling 2060; polyclonal rabbit anti-human PKCζ, 1:1000, Cell Signaling 9372); washed for 10 min with TPBS (twice), 5 min with PBS, followed by a 1 h incubation of appropriate secondary antibodies conjugated to horseradish peroxidase. Equal protein loading was confirmed by reprobing each membrane with calsequestrin monoclonal IgG (Thermo-Scientific PA1-903, 1:2500, polyclonal rabbit anti-dog).

### Quantification of GLUT Translocation to the Cell Surface

Isolated cardiac myocytes were photolabeled with the cell-surface-impermeant biotinylated bis-glucose photolabeling reagent (bio-LC-ATB-BGPA, 300 μM, Toronto Research Chemicals, Toronto, ON, Canada), of which the hexose group interacts specifically with the extracellular binding site of GLUTs. The photolabeled reagent was cross-linked to cell surface GLUTs using a Rayonet photochemical reactor (340 nM, Southern New England UV), as previously described (Waller et al., 2011b, 2013; Maria et al., 2015). Cells were then lysed with 1:500 concentration of RIPA (Thermo Fisher) and Protease Inhibitor Cocktail (Sigma-Aldrich, P8340) and the total lysate was stored in −80°C. Recovery of photolabeled (cell surface) GLUTs from total cardiac membranes (200 μg) was achieved using streptavidin isolation (bound to 6% agarose beads) to facilitate separation of non-cell surface GLUTs (“unlabeled” or intracellular fraction that remains in the supernatant) from cell surface GLUTs (“labeled” or sarcolemmal fraction). The labeled GLUTs were then dissociated from the streptavidin prior to SDS-PAGE and subsequent immunoblotting with GLUT antibody, as previously described (Waller et al., 2013, 2015; Maria et al., 2015).

### Statistical Analysis

Differences between means were assessed using Student’s *t*-tests. Repeated measures two- or one-way ANOVA was performed with Student–Newman–Keuls *post hoc* test for *in vivo* and *in vitro* measurements, respectively. If not normally distributed, the data was analyzed with a Mann–Whitney or Friedman test. Statistical significance was defined as *P* < 0.05. Data are presented as mean ± SE.

## Results

### Acute GGF2 Treatment Increases Glucose Uptake and GLUT4 Translocation in Healthy Adult Cardiac Myocytes

**FIGURE 1:**
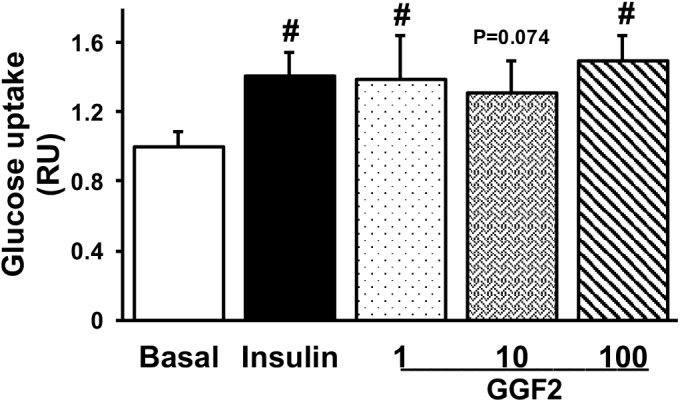
Acute treatment with glial growth factor 2 (GGF2) stimulates glucose uptake in isolated cardiac myocytes from healthy adult rats. Mean ± SE of glucose uptake; values normalized to basal (*n* = 6/group); ^#^*P* < 0.05 relative to basal. Methods: A non-radioactive assay was performed using 2-NDBG in isolated cardiac myocytes following incubation without (i.e., basal) or with insulin or incremental dose of GGF2 (i.e., 1, 10, and 100 ng/ml). RU: relative units.

To determine whether acute *in vitro* GGF2 treatment modulates cardiac glucose transport, we performed a glucose uptake assay in isolated cardiac myocytes from healthy adult rats incubated without (basal) or with insulin or incremental doses of GGF2 prior to incubation with 2-NBDG. Similar to insulin, GGF2 (both at 1 ng/ml and 100 ng/ml) induced an increased glucose uptake in healthy cardiac myocytes (by 40 and 49%, respectively, *P* = 0.002, Figure 1). A trend (*P* = 0.074) toward an increase in glucose uptake was also noted when healthy myocytes were incubated with 10 ng/ml of GGF2.

**FIGURE 2:**
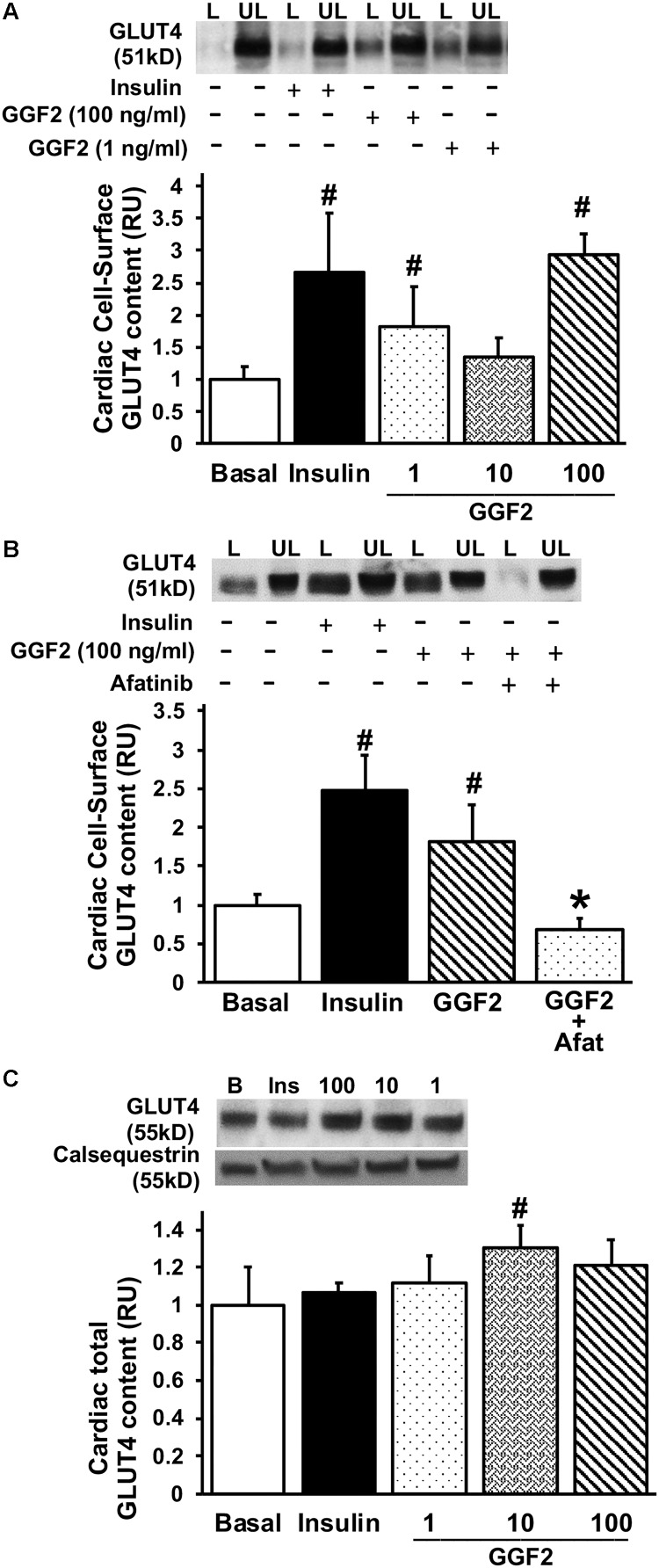
Acute treatment with GGF2 stimulates the regulation of glucose transport via the ErbB receptors in healthy adult cardiac myocytes. **(A)** GGF2 treatment stimulates GLUT4 trafficking to the cell surface. Top panel: representative Western blot. Bottom Panel: Mean ± SE of cell surface GLUT4 protein content; values normalized to basal (*n* = 3-8/group); ^#^*P* < 0.05 vs. basal. Methods: Photolabeled biotinylated assay in isolated rat ventricular myocytes incubated without (i.e., basal, −) or with insulin (+) or incremental dose of GGF2 (i.e., 1, 10, and 100 ng/ml). L, Labeled (cell surface fraction); UL, unlabeled (intracellular fraction). **(B)** ErbB receptor blockade (afatinib) blunts GGF2-stimulated GLUT4 translocation. Top panel: representative Western blot. Bottom Panel: Mean ± SE of cell surface GLUT4 protein content; values normalized to basal (*n* = 3–8/group); ^#^*P* < 0.05 vs. basal; ^∗^*P* < 0.05 vs. GGF2. Methods: Photolabeled biotinylated assay in isolated rat ventricular myocytes incubated without (i.e., basal, −) or with (+) afatinib for 1 h prior to incubation with insulin or GGF2 (100 ng/ml) for 1 h. **(C)** GGF2 increases total GLUT4 expression. Top panel: representative Western blot. Bottom panel: Mean ± SE of protein expression (values expressed relative to basal; *n* = 11–15/group); ^#^*P* < 0.05 vs. basal. Methods: Western blotting from total lysate of isolated rat ventricular myocytes incubated without (i.e., basal) or with insulin or incremental dose of GGF2. Calsequestrin was used as a loading control. RU, relative units.

To measure GLUT4 trafficking, the rate-limiting step in glucose uptake, we performed the state-of-the-art biotinylation assay that specifically quantifies cell membrane GLUT protein content in photolabeled myocytes. Similar to the effect of insulin, GGF2 treatment (both 1 and 100 ng/ml) increased GLUT4 translocation in treated healthy myocytes (by 99 and 184%, respectively, *P* < 0.05) compared to basal levels (Figure 2A,B). We further noted a significant increase in total GLUT4 protein expression when myocytes were incubated with 10 ng/ml of GGF2 (Figure 2C). Since incubating ventricular myocytes with 100 ng/ml of GGF2 induced an increase in GLUT4 trafficking by ∼2 fold (*P* < 0.05), this concentration was selected for subsequent experiments. We then incubated myocytes with afatinib prior to incubation with GGF2. As expected, the GGF2-induced increased GLUT trafficking was blunted when healthy myocytes were incubated with ErbB (2 and 4) blocking antibodies (Figure 2B). These findings confirmed that GGF2 enhanced GLUT4 trafficking to the cell surface of healthy adult cardiac myocytes by activating the ErbB 2/4 receptors.

### Acute GGF2 Treatment Stimulates the Activation of PDK-1, Akt, AS160, and PKCζ in Healthy Adult Cardiac Myocytes

**FIGURE 3:**
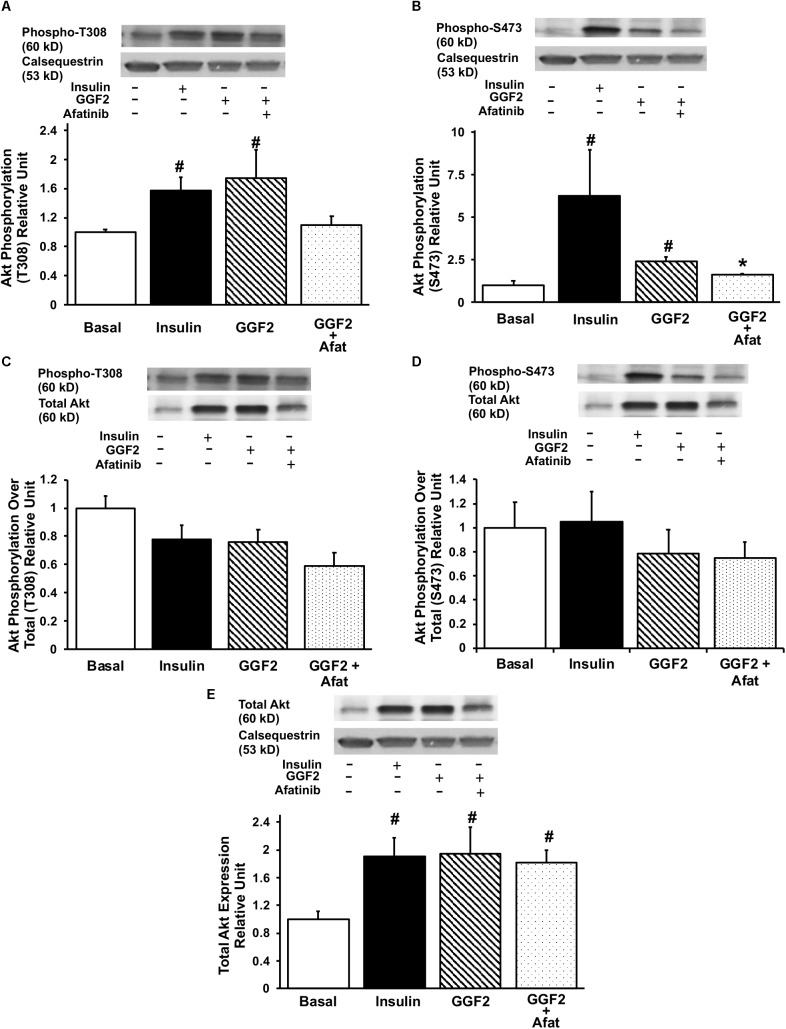
GGF2 treatment enhances Akt phosphorylation at T308 and S473 sites in healthy ventricular myocytes, which is blunted by ErbB receptor blockade (afatinib). Top panels: representative Western blot. Bottom panels: Mean ± SE of protein expression (values expressed relative to basal; *n* = 3–4/group), normalized to calsequestrin **(A,B)** or total expression **(C,D)**; ^#^*P* < 0.05 vs. basal; ^∗^*P* < 0.05 vs. GGF2. **(E)** Total Akt protein expression upon insulin and GGF2 treatment of ventricular myocytes. Top panel: representative Western blot. Bottom panel: Mean ± SE of protein expression (values expressed relative to basal; *n* = 4–5/group); ^#^*P* < 0.05 vs. basal. Methods: Western blotting from total lysate of isolated rat ventricular myocytes incubated without (i.e., basal, −) or with (+) insulin or GGF2 (100 ng/ml). For **(A–E)**, the same membrane was probed for the indicated proteins, with calsequestrin used as the loading control.

In order to investigate the mechanisms regulating GGF2 induced-GLUT trafficking, we then quantified the expression of key downstream effectors (i.e., Akt, PI3K downstream effectors and AS160) by incubating myocytes without (i.e., basal) and with insulin, afatinib or GGF2. Insulin increased phosphorylation of Akt at both T308 and S473 sites (by 157 and 635% compared to baseline, *P* = 0.008 and *P* = 0.034, respectively, Figure 3A,B). Similar to the effects of insulin, we demonstrated that GGF2 significantly increased Akt phosphorylation at both T308 and S473 sites (by 75 and 139% compared to baseline, *P* = 0.029 and *P* = 0.01, respectively, Figure 3A,B), in addition to an increase of total Akt protein expression (by 94% compared to baseline, *P* = 0.043, Figure 3E). In addition, afatinib significantly attenuated GGF2 stimulation of Akt phosphorylation (at T308 and S473 sites), suggesting that activation of ErbB receptors by GGF2 enhances glucose transport in the myocardium through an Akt dependent mechanism (Figure 3A,B). Since total Akt protein significantly increased following GGF2 treatment, there is no significant alteration of Akt phosphorylation at either the T308 or S473 sites when normalized to total Akt (Figure 3C,D).

**FIGURE 4:**
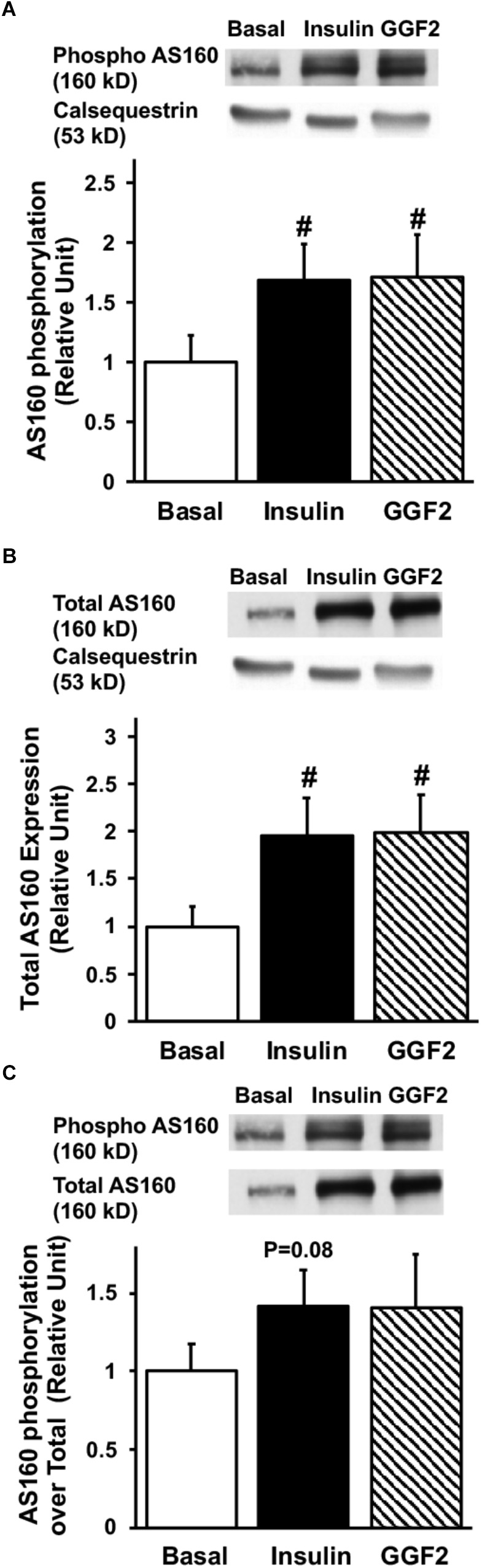
Similar to insulin, acute GGF2 treatment stimulates the phosphorylation **(A)** and total protein expression **(B)** of AS160 (Akt substrate at 160 k) in healthy ventricular myocytes. Top panels: representative Western blot. Bottom panels: Mean ± SE of phosphorylated protein expression (values expressed relative to basal), normalized to calsequestrin **(A)** or total protein expression **(C)**; *n* = 14–17/group; ^#^*P* < 0.05 vs. basal. Methods: Western blotting from total lysate of isolated rat ventricular myocytes incubated without (i.e., basal) or with insulin or GGF2 (100 ng/ml). For **(A–C)**, the same membrane was probed for the indicated proteins, with calsequestrin used as the loading control.

**FIGURE 5:**
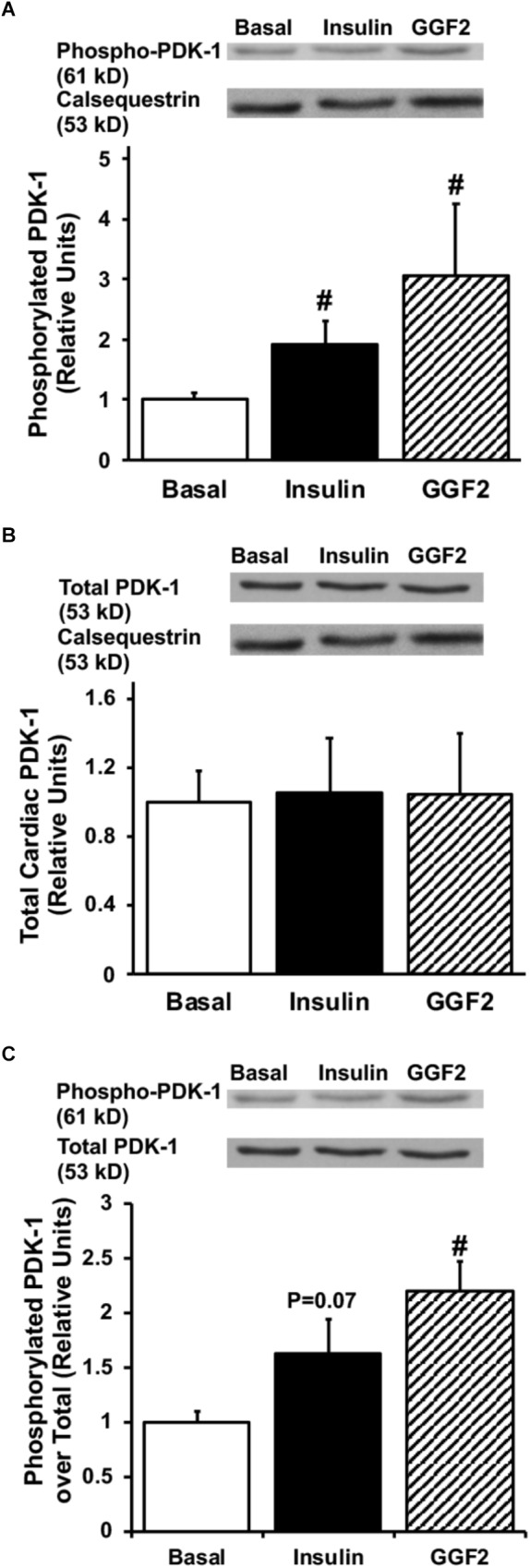
Similar to insulin, acute GGF2 treatment stimulates phosphorylation of PDK-1 in healthy ventricular myocytes. Top panels: representative Western blot. Bottom panels: Mean ± SE of phosphorylated protein expression (values expressed relative to basal), normalized to calsequestrin **(A)** or total protein expression **(C)**; *n* = 4–9/group; ^#^*P* < 0.05 vs. basal. Methods: Western blotting from total lysate of isolated rat ventricular myocytes incubated without (i.e., basal) or with insulin or GGF2 (100 ng/ml). **(B)** Total protein expression of PDK-1 upon insulin and GGF2 treatment of ventricular myocytes. Top panels: representative Western blot. Bottom panels: Mean ± SE of protein expression (values expressed relative to basal); *n* = 7–17/group; ^#^*P* < 0.05 vs. basal. Methods: Western blotting from total lysate of isolated rat ventricular myocytes incubated without (i.e., basal) or with insulin or GGF2 (100 ng/ml). For **(A–C)**, the same membrane was probed for the indicated proteins, with calsequestrin used as the loading control.

**FIGURE 6:**
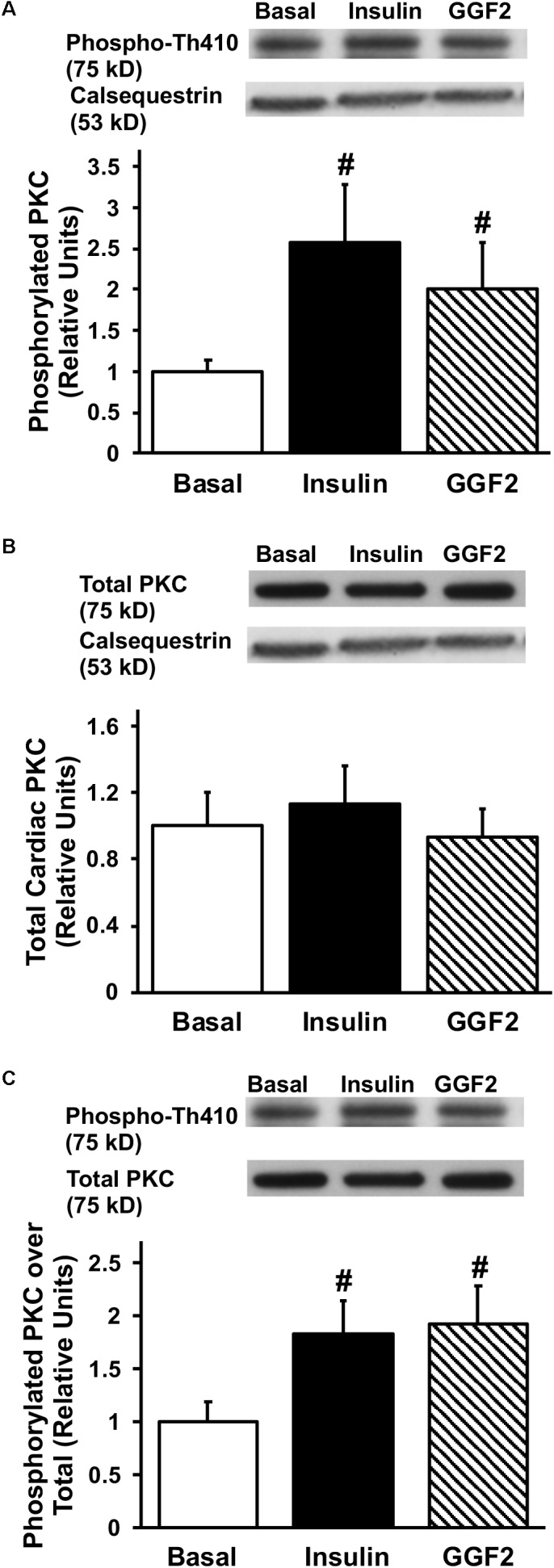
Similar to insulin, acute GGF2 treatment stimulates phosphorylation of PKC in healthy ventricular myocytes. Top panels: representative Western blot. Bottom panels: Mean ± SE of phosphorylated protein expression (values expressed relative to basal), normalized to calsequestrin **(A)** or total protein expression **(C)**; *n* = 4–9/group; ^#^*P* < 0.05 vs. basal. Methods: Western blotting from total lysate of isolated rat ventricular myocytes incubated without (i.e., basal) or with insulin or GGF2 (100 ng/ml). **(B)** Total protein expression of PKC upon insulin and GGF2 treatment of ventricular myocytes. Top panels: representative Western blot. Bottom panels: Mean ± SE of protein expression (values expressed relative to basal); *n* = 7–17/group; ^#^*P* < 0.05 vs. basal. Methods: Western blotting from total lysate of isolated rat ventricular myocytes incubated without (i.e., basal) or with insulin or GGF2 (100 ng/ml). For **(A–C)**, the same membrane was probed for the indicated proteins, with calsequestrin used as the loading control.

Since activation of Akt phosphorylates AS160 (the most downstream signaling protein implicated as a key regulator of GLUT trafficking), we investigated whether GGF2 regulates glucose trafficking through an AS160 dependent pathway in the healthy heart. We demonstrated that similar to the effect of insulin, *in vitro* GGF2 treatment significantly enhances AS160 phosphorylation by 72%, as well as total AS160 protein expression, when normalized to calsequestrin (*P* < 0.05, Figure 4A,B). As compared to total AS160, there was no difference in phosphorylation of AS160 between insulin and GGF2 (Figure 4C). We further hypothesized that there will be an increased activation of PI3K downstream effectors, including PDK-1 and PKC, in both insulin- and GGF2-treated cardiac myocytes. Similar to the effect of insulin, GGF2 treatment significantly enhanced the phosphorylation of PDK-1 and PKC (by 118 and 92% when compared to total protein, *P* < 0.05, respectively, Figure 5, 6). Overall, these data suggest that GGF2 regulates GLUT translocation in healthy cardiac myocytes through an Akt/PKC dependent pathways.

### Acute GGF2 Treatment Partially Rescues Glucose Trafficking During Myocardial Infarction

**FIGURE 7:**
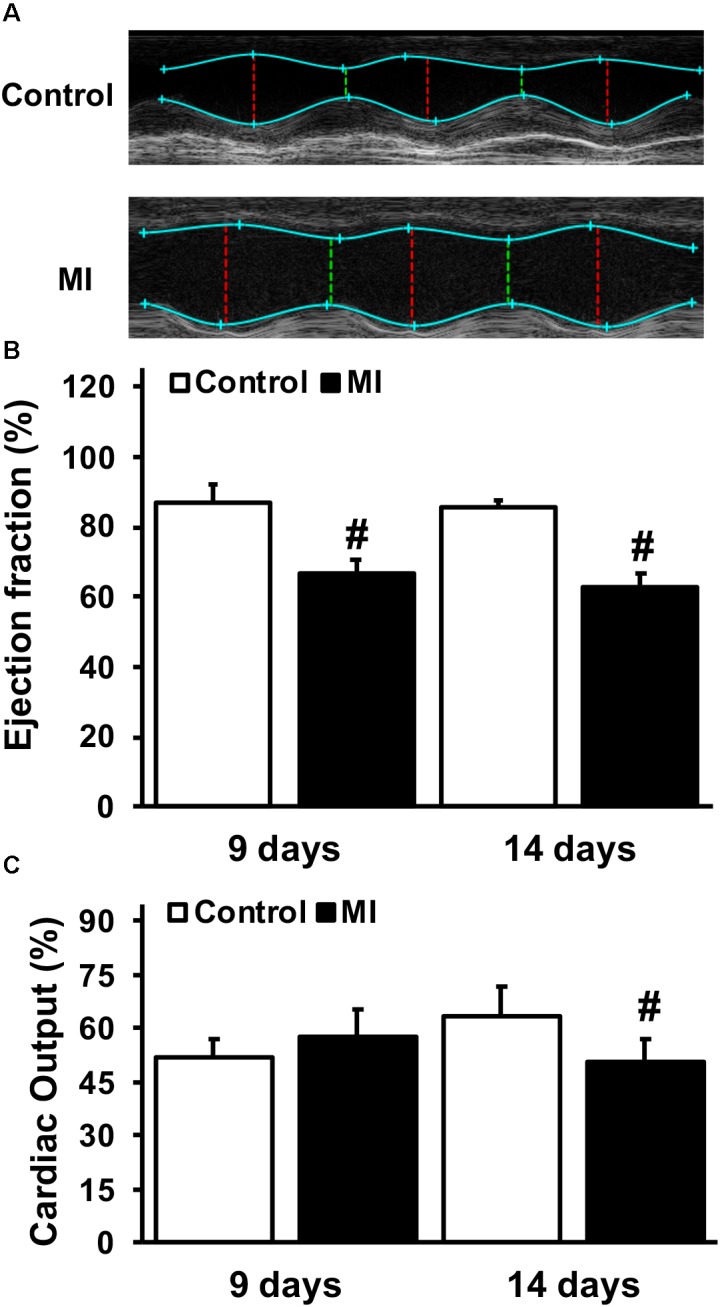
Impaired systolic function and cardiac output during myocardial infarction (MI). **(A)** Representative paired M-mode echocardiograms of age-matched control and MI rats 14 days after left anterior descending coronary artery ligation. **(B)** Mean ± SE of % ejection fraction. **(C)** Mean ± SE of cardiac output (ml/min) (*n* = 2–5/group), ^#^*P* < 0.05 vs. age-matched controls.

The effect of GGF2 on glucose transport was subsequently evaluated in myocytes isolated from rats in which MI was induced by ligating the left anterior descending coronary artery. As expected, ejection fraction, a surrogate of systolic function, was significantly decreased in the MI group at 9 and 14 days after surgery compared to the control groups (*P* < 0.05, Figure 7A,B). In addition, cardiac output was significantly decreased 14 days after surgery, confirming that the MI rats had significant impairment in systolic function (*P* = 0.045; Figure 7C). Heart rate was not significantly different between groups (332.7 bpm ± 2.8, and 333.8 bpm ± 2.5, at day 14 in control and MI groups, respectively).

**FIGURE 8:**
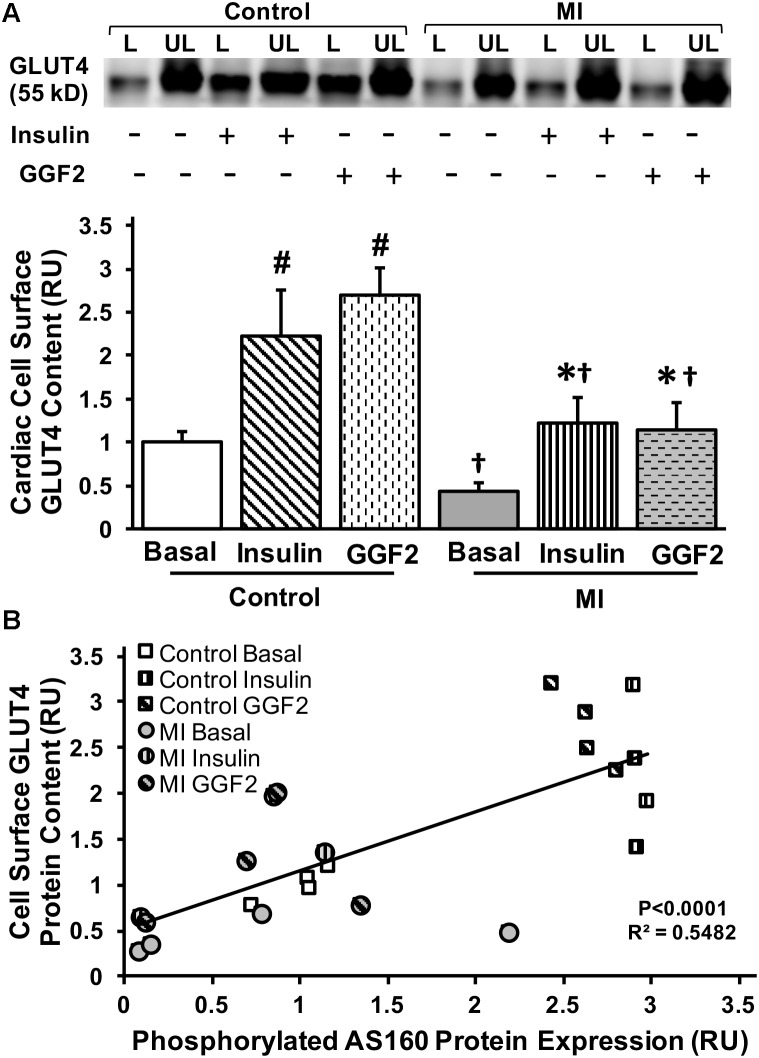
GGF2 treatment partially rescues impaired GLUT trafficking via an AS160 dependent pathway during MI. **(A)** Top panels: representative Western blot. Bottom Panels: Mean ± SE of cell surface GLUT4 protein content in myocytes from MI and age-matched control rats; values normalized to control basal (*n* = 2–4/group); ^#^*P* < 0.05 vs. control basal, ^∗^*P* < 0.05 vs. MI Basal, ^†^*P* < 0.05 vs. same treatment in Controls. Methods: Photolabeled biotinylated assay in isolated rat ventricular myocytes incubated without (i.e., basal, −) or with (+) insulin or GGF2 (100 ng/ml). L, labeled (cell surface fraction); UL, unlabeled (intracellular fraction); Con, control. **(B)** GLUT4 trafficking to the cell surface significantly correlates with AS160 activation in myocytes from healthy and MI rats following incubation with insulin or GGF2. Scatterplot and linear regression of myocardial cell surface GLUT4 content (dependent variable) and AS160 phosphorylation (independent variable) in myocytes of control and MI rats (*n* = 2–4/group) under basal conditions or after *in vitro* insulin or GGF2 treatment; *P* < 0.0001; *R*^2^ = 0.5482; *Y* = 0.6401 *X* + 0.5165. RU, relative units.

We then measured GLUT4 trafficking using the photolabeled biotinylation assay in isolated myocytes from MI and control rats incubated without (basal) or with insulin or GGF2. GLUT4 trafficking was significantly decreased by 44% in MI vs. healthy myocytes under basal conditions (*P* = 0.04). *In vitro* insulin treatment partially rescued GLUT4 trafficking in myocytes from MI rats. Similar to the effect of insulin, *in vitro* GGF2 treatment rescued GLUT4 trafficking in treated MI myocytes compared to untreated MI myocytes (*P* < 0.001, Figure 8A). In addition, we reported a positive linear correlation between cell surface GLUT4 expression and AS160 phosphorylation in myocytes of control and MI rats under basal conditions or after *in vitro* insulin or GGF2 treatment (*P* < 0.0001, *R*^2^ = 0.5482, Figure 8B).

## Discussion

The full-length recombinant GGF2 isoform of NRG-1β is a growth factor which has being explored as a potential therapy for HF in several clinical trials (Brittain et al., 2013; Lenihan et al., 2013). Although GGF2 treatment improves cardiac function after MI in both preclinical studies and clinical trials, the underlying cytoprotective mechanisms in cardiac myocytes are not well known. In this study, we demonstrated that acute *in vitro* GGF2 treatment stimulated GLUT4 translocation, and thus increased glucose uptake, via PDK-1-, Akt-, AS160-, and PKCζ-dependent mechanisms in healthy adult rat cardiac myocytes. Furthermore, we demonstrated that GGF2 treatment partially rescued GLUT translocation in myocytes from MI hearts via an Akt-mediated AS160 phosphorylation.

### Acute GGF2 Treatment Stimulates Glucose Transport via the ErbB Receptors in Healthy Adult Cardiac Myocytes

Being within the EGF family, NRG-1β is a paracrine factor acting on myocytes that regulates both cardiac development and maintenance via the ErbB receptors (Gassmann et al., 1995; Lee et al., 1995; Meyer and Birchmeier, 1995). In this study, we demonstrated that short-term *in vitro* GGF2 treatment increased glucose uptake in healthy adult cardiac myocytes. In agreement with our findings, Cantó et al. (2004) reported that NRG-stimulated glucose transport was additive to insulin in L6E9 myotubes, suggesting the existence of an alternative mechanism to insulin-stimulated glucose uptake. Furthermore, our study utilized GGF2, a full length splice variant of NRG, shown to have therapeutic advantage over rhNRG-1 (EGF-like domain only) in several clinical trials (Brittain et al., 2013; Lenihan et al., 2013).

To sustain continuous pumping action, the energetic demands of the heart are extreme (Ventura-Clapier et al., 2004). Even with its ability to utilize other substrates such as fatty acids, lactate, ketone bodies, and amino acids, the heart utilizes more glucose than skeletal muscle, lung, or adipose tissue in order to maintain homeostasis. Therefore, glucose uptake is crucial to healthy cardiac function (Ware et al., 2011; Waller et al., 2013, 2015). Glucose uptake, the rate-limiting step in whole-body glucose homeostasis, is regulated by a family of specialized proteins, called the GLUTs. GLUT4 is the major isoform expressed in myocytes and is translocated from an intracellular pool to the cell membrane (active site) following insulin stimulation. Under basal conditions, GLUT4 is primarily located within multiple intracellular compartments such that only 2–5% is observed at the plasma membrane (Klip et al., 2014). The molecular mechanisms regulating glucose transport in the myocardium are still not well elucidated. One of the challenges in studying cardiac metabolism in murine models is the quantification of cell surface GLUTs. Therefore, as the translocation of GLUT4 precedes glucose uptake in insulin-sensitive tissues, (Klip et al., 2014) we evaluated GLUT4 trafficking by the state-of-the-art biotinylation photolabeled assay that quantifies both the protein content at the cell membrane and the intracellular GLUT pool (Waller et al., 2013, 2015; Maria et al., 2015). Using this technique, our results demonstrated that short-term GGF2 treatment stimulated GLUT4 translocation in adult cardiac myocytes to the same extent as insulin. Similar to our findings, Suarez et al. (2001) demonstrated a 43% increase in GLUT4 abundance within plasma membrane fractions of skeletal myocytes when treated with NRG. Although GLUT trafficking was not significantly increased when myocytes were incubated with 10 ng/ml of GGF2, we noted a significant increase in total GLUT4 protein expression. These data suggested that GGF2 may modulate glucose transport in healthy myocytes in a biphasic manner, which may be dose-dependent, as previously reported in striated muscle (Florini et al., 1996; Baliga et al., 1999; Kim and Caggiano, 2018). Overall, incubating ventricular myocytes with GGF2 at high concentration (i.e., 100 ng/ml) induced both an increase in GLUT4 trafficking (by ∼3 fold) and total GLUT4 protein expression. As a result, GGF2 increased glucose uptake in healthy myocytes to the same extent as insulin (a potent anabolic hormone).

The pharmacological action of NRG is initiated upon binding to its receptors tyrosine kinase of the EGF receptor family (e.g., ErbB 2/4, both of which are expressed in cardiac myocytes), triggering phosphorylation of the downstream cascade of effectors (Holbro and Hynes, 2004). To evaluate the role of these tyrosine kinase receptors, we incubated myocytes with afatinib (prior to GGF2 treatment), a Akt inhibitor that irreversibly inhibits ErbB2/4 by covalently binding to cysteine number 797 of the ErbB (Li et al., 2008). In alignment with previous studies on incubated soleus muscle, (Cantó et al., 2006) we demonstrated that inhibition of ErbB2 and ErbB4 (by afatinib) blunted NRG-induced GLUT4 translocation in adult cardiac myocytes. In brief, our data demonstrated that acute *in vitro* GGF2 treatment enhanced GLUT4 translocation in healthy cardiac myocytes by activating the ErbB 2/4 receptors, which subsequently increased glucose transport in the myocardium.

### GGF2 Treatment Modulates Glucose Uptake via an Akt-Dependent Pathway in Healthy Adult Cardiac Myocytes

It has been well elucidated that ErbB2 and ErbB4 activation initiates PI3K signaling cascades in different tissues such as skeletal muscle and breast cancer cells (Canfield et al., 2015). Similarly, our results indicated an increase in PDK-1 phosphorylation, a downstream target of PI3K, in response to GGF2 treatment in adult cardiac myocytes. In L6E9 myotubes, PDK-1 has also been demonstrated to be an essential downstream target of PI3K, in the induction of glucose transport by NRG stimulation (Cantó et al., 2004). The marked increase in p-PDK-1 leads us to infer that it is highly sensitive to GGF2 similar to the traditionally accepted activation by insulin (Coffer et al., 1998).

Furthermore, our results showed an increase in phosphorylation of Akt, a downstream target of PDK-1, following GGF2 treatment. In agreement with previous studies, acute insulin treatment increases Akt phosphorylation at both T308 and S473 sites in cardiac myocytes (Long et al., 2011; Maria et al., 2015). Similarly, NRG-1 is known to activate Akt phosphorylation in L6E9 myotubes and soleus muscle (Cantó et al., 2004). In the current study, GGF2 treatment enhanced Akt phosphorylation (at both the serine and threonine sites) while inhibition of the ErbB 2/4 receptors by afatinib diminished GGF2-stimulated Akt activation. Phosphorylation of the T308 site has been attributed to stimulation from the PI3K/PDK1 pathway while phosphorylation of the S473 site has been attributed to the AMPK/MTORC2 pathway (Downward, 1998; Bayascas and Alessi, 2005). Since we reported in the current study a similar increase in phosphorylation at both sites after GGF2 treatment, our data suggest that GGF2 stimulates GLUT4 translocation through these two different signaling pathways (Downward, 1998; Bayascas and Alessi, 2005). We also here reported an upregulation of total Akt protein, as previously reported by other groups after short-term treatment with insulin (Morrison et al., 2011; Gao and Li, 2018). These findings suggest that Akt is an essential downstream target of ErbB2/4 activation in the stimulation of glucose uptake. In addition, upon GGF2 stimulation, we observed a significant increase in the activation of AS160, the downstream effector of Akt.

Lacking stimulation from insulin or calcium, GLUT4 is retained inactively in intracellular pools. The phosphorylation of AS160 is the final step in the downstream insulin-signaling pathway which promotes the activation of RabGTP, which initiates the translocation of the GLUT-containing vesicle to dock and diffuse to the cell surface (Waller et al., 2011a; Ware et al., 2011; Lacombe, 2014). Similar to Akt, we reported a significant upregulation of total AS160 protein expression which may inherently produce an increased amount of phosphorylated protein. Given the similarity between the results in insulin-stimulated and GGF2-stimulated myocytes, it would appear that both of these pharmacological agents work through similar pathways to upregulate AS160. Overall, these data indicated that GGF2 treatment regulates glucose trafficking in cardiac myocytes independently of insulin via the PI3K/AS160 pathway. We further demonstrated an increased expression of phosphorylated PKC-ζ and PDK-1 without increasing their total protein expression. Taken together, these results indicated 2 different mechanisms to activate phosphorylation sites of the downstream signaling pathway (Wu et al., 2009).

Although PKC-ζ, a downstream target of PDK-1, plays a crucial role in glucose uptake, its role in the heart is not well understood. Insulin stimulation rapidly increased PKC-ζ activity in adipocytes and L6 myotubes (Bandyopadhyay et al., 1997). Similarly, in the current study, acute GGF2 treatment significantly increased activation of PKCζ in cardiac myocytes to the same extent as insulin stimulation. This is in contrast with previous reports in which NRG had a stronger maximal effect on PKC than insulin in L6E9 myotubes (Cantó et al., 2004). From these data, we conclude that GGF2 increases glucose uptake via a PKCζ-dependent mechanism in cardiac myocytes, but to a lesser extent than what has been reported in skeletal muscle.

### GGF2 Treatment Partially Rescues Impaired Glucose Transport During Myocardial Infarction

Myocardial infarction, or heart attack, is characterized by the lack of blood flow to the myocardial tissue resulting in damage to, or necrosis of, the cardiac myocytes (Mythili and Malathi, 2015). The effect of MI on cardiac glucose metabolism is somewhat controversial. Doenst and colleagues did not report any effect of MI on glucose uptake or Akt activation at 2 weeks post-MI compared to controls (Amorim et al., 2010). In contrast, several studies indicated that chronic HF decreased glucose uptake and utilization, secondary to cardiac insulin resistance (Murray et al., 2006; Penuelas et al., 2007). For instance, microPET imaging after MI indicated a moderate to severe reduction in glucose uptake in the apex, apical anterior and apical lateral segments as early as 48 h post infarct (Penuelas et al., 2007). In addition, insulin-stimulated glucose uptake was reduced by 42% in isolated, perfused, infarcted hearts, in parallel with a 28% decrease in total GLUT4 expression (Murray et al., 2006). In agreement with these findings, we reported a significant decrease in GLUT4 trafficking in isolated myocytes 14 days post-MI, which suggests that MI induces a state of cardiac insulin resistance.

With the rate of glucose utilization being greater in the heart compared to other tissues, altered substrate utilization, resulting from decreased glucose uptake and/or oxidation, could underlie contractile dysfunction reported during HF (Ware et al., 2011; Lacombe, 2014). It has been suggested that failure to utilize glucose oxidation as an energy source increases fatty acid oxidation within the mitochondria and subsequent accumulation of reactive oxygen species, which may contribute to cardiac dysfunction and MI (Povlsen et al., 2013). Several studies have demonstrated the cardioprotective effect of enhanced glucose uptake and utilization by the heart (Sodi-Pallares et al., 1962). For instance, the heart is better protected against MI in the fed state compared to the fasted state, since myocytes are able to utilize more glucose as an energy source (Liepinsh et al., 2014). In addition, cardiac-specific GLUT1 overexpression provided protection against the aging-associated increase of susceptibility to MI (Luptak et al., 2007). Glucose–insulin–potassium (GIK) therapy has also been shown to be cardioprotective (Sodi-Pallares et al., 1962). It has been proposed that optimizing energy substrate metabolism by decreasing fatty acid β-oxidation, while increasing the rates of glycolysis and glucose oxidation, will enhance ATP production and utilization efficiency, and therefore restore cardiac efficiency in the ischemic/reperfused failing hearts (Jaswal et al., 2011). Furthermore, the shift toward glucose utilization has been correlated with decreased infarct size and improved post-ischemic cardiac function (Zhang et al., 2006). Therefore, metabolic therapy has emerged as a promising therapeutic strategy for patients with MI and HF. Interestingly, while a downregulation of the ErBB receptors has been reported in human patients in HF (Rohrbach et al., 2005). NRG-1β is released in response to ischemia and stress, and activates the ErbB2/ErbB4 receptors in a paracrine manner (Zhao et al., 1998). In addition, treatment with the GGF2 improved cardiac function in MI-induced systolic dysfunction (Hill et al., 2013). Importantly, we demonstrated that acute *in vitro* GGF2 treatment partially rescued alterations in GLUT4 trafficking during MI. We further reported a positive linear correlation between GLUT4 trafficking and AS160 activation when myocytes from healthy and MI rats were incubated with insulin or GGF2. Since the translocation of GLUT4 to the plasma membrane precedes glucose transport, (Klip et al., 2014) these findings suggested that, similar to our data in the healthy myocardium, GGF2 increased glucose uptake via an Akt-dependent pathway in myocytes from infarcted hearts.

## Conclusion

We demonstrated that acute treatment with the recombinant GGF2 isoform of NRG-1β increased glucose transport in cardiac myocytes by activating the ErbB 2/4 receptors and subsequent key downstream effectors (i.e., PDK-1, Akt, AS160, and PKC). We further demonstrated that GGF2 partially rescued GLUT4 translocation during MI via Akt-mediated AS160 phosphorylation. Since increased glucose uptake has been shown to be cardio-protective during MI, (Sodi-Pallares et al., 1962; Zhang et al., 2006; Jaswal et al., 2011; Hill et al., 2013; Povlsen et al., 2013; Liepinsh et al., 2014) insights gained from this study identified novel mechanisms of actions by which GGF2 could confer cardioprotection during MI. Therefore, better understanding of the regulation of GGF2-stimulated glucose transport and utilization may lead to its use as a novel therapeutic target for the treatment of patients with HF.

## Disclosures

Acorda Therapeutics provided both the compound and the funding.

## Author Contributions

VL conceived and designed the experiments. SS, ZM, NR, AC, and DM performed the experiments. SS, ZM, NR, AC, DM, and VL analyzed and interpreted the data. SS, VL, AC, ZM, and PL wrote and/or edited the manuscript.

## Conflict of Interest Statement

The authors declare that the research was conducted in the absence of any commercial or financial relationships that could be construed as a potential conflict of interest.

## Abbreviations

Akt: protein kinase B
AS160: AKT substrate at 160 kDa
EGF: epidermal growth factors
GGF2: glial growth factor 2
GLUT: glucose transporter
HF: heart failure
MI: myocardial infarction
NRG: neuregulin
PDK-1: phosphoinositide-dependent kinase -1
PI3K: phosphoinositide 3-kinase
PKC: protein kinase C.
